# Quantitative assessment of rest and acetazolamide CBF using quantitative SPECT reconstruction and sequential administration of ^123^I-iodoamphetamine: comparison among data acquired at three institutions

**DOI:** 10.1007/s12149-014-0879-9

**Published:** 2014-07-08

**Authors:** Miho Yamauchi, Etsuko Imabayashi, Hiroshi Matsuda, Jyoji Nakagawara, Masaaki Takahashi, Eku Shimosegawa, Jun Hatazawa, Michiyasu Suzuki, Hideyuki Iwanaga, Kenji Fukuda, Koji Iihara, Hidehiro Iida

**Affiliations:** 1 Department of Investigative Radiology, National Cerebral and Cardiovascular Center Research Institute, 5-7-1 Fujishiro-dai, Suita, Osaka 565-8565 Japan; 2 Department of Nuclear Medicine, Saitama Medical University, 1397-1 Yamane, Hidaka, Saitama 350-1298 Japan; 3 Nakamura Memorial Hospital, 2 Kawazoe, Minami, Sapporo, Hokkaido 005-0802 Japan; 4 Department of Nuclear Medicine, Osaka University School of Medicine, 2-2 Yamadaoka, Suita, Osaka 565-0871 Japan; 5 Department of Neurosurgery, Yamaguchi University School of Medicine, 1-1-1 Minami-Kogushi, Ube, Yamaguchi 755-8505 Japan; 6 Department of Radiological Technology, Yamaguchi University Hospital, 1-1-1 Minami-Kogushi, Ube, Yamaguchi 755-8505 Japan; 7 Department of Neurosurgery, National Cerebral and Cardiovascular Center Hospital, 5-7-1 Fujishiro-dai, Suita, Osaka 565-8565 Japan; 8 Present Address: Integrative Brain Imaging Center, National Center of Neurology and Psychiatry, 4-1-1 Ogawa-Higashi, Kodaira, Tokyo 187-8551 Japan; 9 Present Address: Department of Neurosurgery, Integrative Stroke Imaging Center, National Cerebral and Cardiovascular Center Hospital, 5-7-1 Fujishiro-dai, Suita, Osaka 565-8565 Japan; 10 Present Address: Department of Neurosurgery, Fukuoka University School of Medicine, Fukuoka, Kyushu 814-0180 Japan; 11 Present Address: Department of Neurosurgery, Kyushu University School of Medicine, Fukuoka, Kyushu 812-8582 Japan

**Keywords:** Single-photon emission computed tomography, Cerebral blood flow, Cerebral vascular disease, Kinetic modeling, Ischemia

## Abstract

**Purpose:**

A recently developed technique which reconstructs quantitative images from original projection data acquired using existing single-photon emission computed tomography (SPECT) devices enabled quantitative assessment of cerebral blood flow (CBF) at rest and after acetazolamide challenge. This study was intended to generate a normal database and to investigate its inter-institutional consistency.

**Methods:**

The three institutions carried out a series of SPECT scanning on 32 healthy volunteers, following a recently proposed method that involved dual administration of ^123^I-iodoamphetamine during a single SPECT scan. Intra-institute and inter-institutional variations of regional CBF values were evaluated both at rest and after acetazolamide challenge. Functional images were pooled for both rest and acetazolamide CBF, and inter-institutional difference was evaluated among these images using two independent software programs.

**Results:**

Quantitative assessment of CBF images at rest and after acetazolamide was successfully achieved with the given protocol in all institutions. Intra-institutional variation of CBF values at rest and after acetazolamide was consistent with previously reported values. Quantitative CBF values showed no significant difference among institutions in all regions, except for a posterior cerebral artery region after acetazolamide challenge in one institution which employed SPECT device with lowest spatial resolution. Pooled CBF images at rest and after acetazolamide generated using two software programs showed no institutional differences after equalization of the spatial resolution.

**Conclusions:**

SPECT can provide reproducible images from projection data acquired using different SPECT devices. A common database acquired at different institutions may be shared among institutions, if images are reconstructed using a quantitative reconstruction program, and acquired by following a standardized protocol.

## Introduction

Single-photon emission computed tomography (SPECT) can provide valuable diagnostic information in clinical patients with various cerebral diseases including cerebral vascular diseases, cognitive disorders, and others. Recently, computer-aided diagnosis-supporting tools have been shown to be of use, to highlight regions or image-pixels that are significantly different from previously determined normal database in the stereotactic domain. Minoshima et al. [1] developed a program, called the three-dimensional stereotactic surface projection (3D-SSP), and it was first applied to identify unique patterns suggestive of Alzheimer’s disease for ^18^F-fluorodeoxy glucose positron emission tomography (PET) images [2, 3]. *Z* values were referred to identify regions which are different as compared to the normal database by means of the statistical analysis procedures. This software has been extended to SPECT images obtained with ^123^I-labeled cerebral perfusion tracer (^123^I-iodoamphetamine, ^123^I-IMP) to detect suggestive defect of cerebral blood flow (CBF) (magnitude and the extent of the defect) in probable Alzheimer disease patients [4]. The 3D-SSP software has further been applied to rest- and acetazolamide-CBF images quantitatively assessed with ^123^I-IMP and SPECT. The severity of hemodynamic cerebral ischemia was then classified into the 3 stages (Stage 0–II) [5], depending on the absolute CBF at rest and % increase of CBF after acetazolamide challenge (cerebral vascular reactivity, CVR) [6, 7]. Matsuda et al. [8] also developed another software package, called easy *Z* score imaging system (eZIS), which involved the normalization of volumetric images to a standard atlas, and the pixel-based statistical analysis, by employing a part of functions of the statistical parametric mapping (SPM) software of Friston et al. [9]. Compensation for inter-institutional differences of SPECT images was implemented in this software package, using a 3D-Hoffman phantom data. The package was applied to cerebral perfusion studies with ^99m^Tc-ethylcysteinate dimer (^99m^Tc-ECD) acquired at 4 institutions using different SPECT cameras, demonstrating the significant discrimination of Alzheimer disease patients from the control group images.

Usage of computer-aided diagnosis-supporting tools is beneficial in many situations, not only for clinical research but also in some of routine clinical diagnosis. Sharing a common database acquired at different institutions would, however, be a challenging issue in SPECT, and the inter-equipment consistency of SPECT images is not well supported. In one example multicenter evaluation demonstrated that images reconstructed using the installed software programs in SPECT devices of different vendors are so different, even though the same projection data are provided [10, 11]. This is attributed to lack of standardized image reconstruction procedures, including different specification of the program coding, and also different procedures to correct for attenuation and scatter. Use of 3D-Hoffman brain phantom images may improve the inter-institutional consistency, which has been proposed by Matsuda et al. [8], but its contribution has not been evaluated yet fully, under the circumstances where attenuation maps are given from the emission projection rather than the transmission scan, and errors attributed to this are not taken into account in the 3D-Hoffman phantom experiment.

More recently, a novel software package for quantitative SPECT reconstruction (QSPECT) has been developed, which can reproduce the radioactivity distribution from original projection data acquired using standard, commercially available SPECT systems [12, 13]. This software was then applied to a clinical protocol of assessing rest- and acetazolamide-CBF images, and demonstrated that regional CBF images agreed well with results from PET technique both at rest and after acetazolamide challenge [13, 14]. It was also shown that the quantitative CBF values were reproducible between the first and the second scans within a month interval, in 44 patients at 9 institutions [13]. Further, Yoneda et al. [15] demonstrated that quantitative CBF and CVR obtained from the same patients repeatedly acquired using different SPECT systems equipped at different institutions were reproducible, both in the quantitative regional values and also in relative distributions. These findings suggested that SPECT images can be reproducible among different SPECT systems installed at different institutions, provided that the clinical protocols are well standardized for CBF quantitation. A database or reference images acquired using different SPECT devices at different institutions may therefore be shared, suggesting the feasibility of using computer-aided diagnosis-supporting tools. Although the SPECT has been considered to be able to provide images intrinsically independent of the geometric design of the cameras [16], this is a new concept which needs to be confirmed.

This study is intended to evaluate the inter-institutional agreement quantitative CBF values at rest and after acetazolamide challenge obtained at three independent institutions. Three sets of normal database were generated at three institutions according to a previously validated protocol of the ^123^I-IMP dual-table autoradiograph combined with the quantitative reconstruction software (QSPECT/DTARG) (http://www.qspect.org) [13–15]. We evaluated inter-institutional consistency of regional CBF values among the three institutions. We also tested the inter-institutional consistency of functional CBF images normalized at stereotactic domain, using two independent software programs of 3D-SSP [1, 5] and SPM [8, 9].

## Materials and methods

### Institutions

Three institutions listed in Table 1 participated in this study. All institutions were equipped for SPECT scans for quantitative assessment of rest- and acetazolamide-CBF using ^123^I-IMP according to the DTARG protocol [13, 14]. QSPECT/DTARG software package [13] was installed in all institutions. SPECT cameras are from two different vendors, and each fitted with different sets of collimators (Table 1).

**Table 1:** Study conditions at three institutions

Institution	SPECT camera	Collimator^a^	BCF^b^ (Bq/mL)	Well/SPECT CCF^c^	Spatial resolution^d^ (mm)	# of subjects	Age^‖^ (years)	Weight (kg)	Administration dose of acetazolamide^¶^ (mg/kg)	PaCO_2_ (mmHg)	Administration dose of^123^I-IMP** (MBq)
A	SIEMENS Symbia	LMEGP parallel beam	87956	0.799	17.13	*N* = 9 (*M* = 4, *F* = 5)	63.9 ± 2.5	57.4 ± 7.8	17.8 ± 2.4 (total dose fixed at 1000)	38.5 ± 4.5	122.1 ± 3.3
B	TOSHIBA GCA9300	LMEHR fan beam (N2)	72281	0.619	11.22	*N* = 13 (*M* = 5, *F* = 8)	59.1 ± 2.5	62.8 ± 11.3	15.0 ± 0.1	40.2 ± 0.7	142.2 ± 8.4
C	TOSHIBA GCA9300	LESHR fan beam (N1)	112419	0.948	10.26	*N* = 10 (*M* = 2, *F* = 8)	63.2 ± 4.2	50.1 ± 7.8	16.8 ± 0.9	37.5 ± 3.1	155.9 ± 14.9
Total	–	–	–	–	–	*N* = 32 (*M* = 11, *F* = 21)	61.7 ± 3.8	57.4 ± 10.7	16.4 ± 1.9	38.8 ± 3.3	139.8 ± 16.1

Results from multiple comparison test: ^‖^ *P* < 0.05 for A vs. B, ^¶^ *P* < 0.05 for A vs. B and B vs. C, ** *P* < 0.05 for A vs. B, B vs. C and A vs. C

^a^Collimator: *LMEGP* low-medium energy general purpose, *LMEHR* low-medium energy high resolution, *LESHR* low energy super-high resolution

^b^BCF: Becquerel calibration factor is the factor to convert reconstructed images by QSPECT to have units of Bq/mL. Smaller number corresponds to greater sensitivity of SPECT system

^c^CCF: cross-calibration factor is defined as the sensitivity of well counter relative to SPECT images. Since SPECT images are already converted to Bq/mL, CCF corresponds to the absolute sensitivity to given radioisotopes

^d^Spatial resolution was determined from the experiment using the 3-dimensional brain phantom (see text)

### Subjects

In total, 32 healthy volunteers whose age ranged from 55 to 70 were selected for the SPECT study. Gender, age and other characterization are shown in Table 1. Age was a significant difference between institutions A and B (Steel–Dwass test, *P* < 0.05) despite of only small difference of 4.8 yo, attributed to the small range of the age in these two institutions. Weight and partial pressure of carbon dioxide in arterial blood (PaCO_2_) showed no significant difference among three institutions. Screening of normal status included a medical review of past history, a physical examination and neurological and mental tests. Subjects having past history of hypertension, diabetes mellitus or atrial fibrillation were excluded. The laboratory studies included a complete blood counts to screen for hematological diseases; serum electrolytes (Na, K, Cl), creatinine and blood urea nitrogen to screen for renal disease; fasting blood glucose, total cholesterol, triglyceride and uric acid to screen for metabolic disease; and total plasma protein and albumin, bilirubin, alkaline phosphatase, serum glutamic oxaloacetic transaminase and γ-glutamyl transpeptidase to screen for liver disease. Before the SPECT study, unenhanced computed tomography (CT) or anatomical magnetic resonance imaging system (MRI) scan was performed to rule out organic lesions of the brain. Subjects with leukoaraiosis and/or asymptomatic lacunar infarction were excluded.

The study protocol was approved by the ethics committees at each institution that followed the principles of the Declaration of Helsinki, and also approved by National Cerebral and Cardiovascular Center for the core labo analysis. All subjects gave written informed consent before the SPECT scan at each institution.

### SPECT scan

A series of experiments was carried out on phantoms prior to the clinical study at each institution, to determine parameters necessary for the quantitative reconstruction using the QSPECT/DTARG software [13] and also to confirm the quality of SPECT images. The collimator septal penetration from high-energy photons into the primary 159-keV energy window for ^123^I was implemented as a part of the scatter correction processes as described previously [12], and was determined using a line source of ^123^I-solution placed at the center of a uniform cylindrical phantom [13, 17]. A factor to calibrate the reconstructed image to the absolute radioactivity concentration in units of Bq/mL (Becquerel calibration factor, BCF) was determined using a ^123^I-radiopharmaceutical syringe (^123^I-iodoamphetamine) of known activity supplied from a radiopharmaceutical company (Nihon Medi-Physics, Tokyo, Japan) [13]. The smaller value of BCF corresponded to the higher sensitivity of SPECT system (Table 1). The cross-calibration factor (CCF) between a well counter and the reconstructed SPECT image was determined using a uniform cylindrical phantom of 16 cm of inner diameter and 15 cm in length filled with the ^123^I-solution [12, 13, 18]. Uniformity and image quality were also confirmed on the SPECT images of the uniform cylindrical phantom reconstructed with QSPECT as described previously [13]. CCF was defined as the sensitivity of well counter relative to SPECT images. Since SPECT images were already converted to Bq/mL, CCF corresponds to the absolute sensitivity to given radioisotopes.

An additional experiment was carried out on a recently developed 3-dimensional Brain Phantom [19], which simulates the CBF distribution in the gray matter regions with realistic head contour and the skull structure. The gray matter compartment was filled with ^123^I-solution of approximately 10 MBq, and the skull compartment with K_2_HPO_4_ solution [19]. SPECT scan followed the same protocol as for the clinical study, and consisted of 7 frames of 4 min, over 28 min. The projection data were summed over the entire scan period, and reconstructed according to the same procedures for clinical data.

**Fig. 1:**
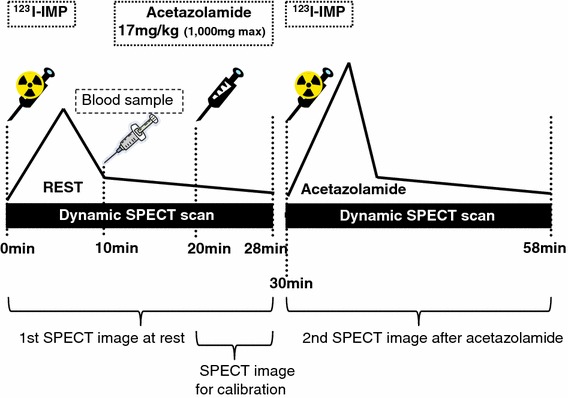
Scanning protocol flow for the DTARG procedures. ^123^I-iodoamphetamine (^123^I-IMP) was injected at 0 min, and 28-min resting dynamic SPECT scan was commenced. Blood sample for calibration of population input function was drawn at 10 min. Acetazolamide was administered at 20 min. CBF values are scaled by last frame (time 24–28 min). Second dynamic SPECT scan followed second injection of ^123^I-IMP at 30 min

The SPECT scan was then carried out on healthy volunteers according to the DTARG protocol, with dual administration of ^123^I-IMP (Fig. 1) [13]. Briefly, 2 dynamic SPECT scans each 28 min recording consisted of 7 frames of 4 min each. ^123^I-IMP (111 or 167 MBq dose calibrated at noon) was infused twice over 1 min into the antecubital vein at 0 and 30 min. Acetazolamide (15–17 mg/kg, see Table 1) was administered intravenously at 20 min after the first ^123^I-IMP injection, corresponding to 10 min before the second ^123^I-IMP injection. The dosage of acetazolamide was determined independently, to follow the routine protocol at each institution (Table 1). A single arterial blood sample was taken at approximately 10 min, and its whole-blood radioactivity concentration was counted using the well counter cross-calibrated to SPECT images. The individual arterial input function was then determined by calibrating a population-based standardized input function using this whole-blood radioactivity concentration [11, 13, 18, 20, 21]. This procedure provides CBF values which agree with those by frequent arterial blood sampling within ±10 % accuracy, with no systematic dependency to the smoking, and the presence of cardiac and/or lung diseases, as described in earlier reports [18, 20, 22, 23].

### Data processing

Projection data from clinical scans were summed for the acquisition duration of the first and second scans, in which uniformity and center-of-rotation corrections were performed using the clinical routine software on the SPECT system. A fan beam-to-parallel beam conversion was applied for data acquired using fan-beam collimators (institutions B and C). The projection data were then transferred to an off-line computer for further analyses.

All images were reconstructed using the QSPECT reconstruction software [13], according to the previously published procedures [12, 14, 17, 24–27]. The software uses a wrapper written in JAVA to run programs written in C for Microsoft Windows systems. A threshold-based edge-detection algorithm generated the attenuation coefficient (*μ*) map, assuming a uniform attenuation coefficient of 0.160 cm^-1^ for ^123^I as an average over the brain and skull regions [12]. The threshold was optimized via the user interface to correctly define the brain outline. The attenuation *μ*-map was generated from the summed 0- to 28-min rest frame and was co-registered to the other images [28] reconstructed with filtered back-projection (FBP) without attenuation or scatter correction. The attenuation *μ*-maps were forward projected to provide the transmission projection data for the transmission-dependent convolution subtraction (TDCS) scatter correction technique. The emission projections were scatter-corrected by the TDCS method, as originally proposed by Meikle et al. [29], and further optimized for realistic data in the brain and thorax regions [14, 26, 27, 30, 31]. An offset compensated for the septal penetration of high-energy photons for ^123^I studies, which adds essentially uniform background counts to the projections. Scatter- and attenuation-corrected images were reconstructed with the ordered-subset the maximum likelihood expectation maximization (MLEM) reconstruction (3 iterations, 5 subsets using geometric-mean projections, post-reconstruction Gaussian filter of 7 mm in full-width at half-maximum) and then realigned to the image set obtained from the first scan. Reconstructed SPECT images are calibrated in Bq/mL, which provides independence from scanning parameters such as the acquisition time, the number of views, a matrix size, and a zoom factor.

Rest- and acetazolamide-CBF images were calculated using the DTARG program, a part of the QSPECT software package, in which additional spatial-smoothing filter of Gaussian with 7-mm full-width at half-maximum (FWHM) was applied. The global CBF over the entire gray matter was estimated from the SPECT frame covering 24–28 min, because this timing minimizes the individual shape variations in individual input function. The look-up table generated for estimating CBF images from the complete dynamic study (0-28 min) was then scaled to provide global cortical gray matter CBF values consistent with the 24- to 28-min frame estimates. A careful detection algorithm was used to reliably exclude extra-cranial accumulation of ^123^I-IMP (e.g., in the parotid region), which could adversely affect this scaling procedure. The regional CBF was then estimated at each pixel by means of the table look-up procedure [12, 18]. The background image at the time of the second ^123^I-IMP injection was estimated from the first-phase CBF images, according to the compartment model assumed in this study [14]. An additional table look-up procedure was applied to the second dynamic dataset (30–58 min) for calculating the vaso-dilated (acetazolamide challenge) CBF images as described previously [13, 14]. The entire procedures for image reconstruction and CBF estimation were carried out at each institution. To facilitate and provide consistent analysis, the data were presented from the reanalysis conducted at the core lab, in which the distribution volume (*V*_d_) of ^123^I-IMP was set to 35.0 mL/mL, a consistent post-filter operation was applied, and the blood density of 1.06 g/mL was appropriately taken into account.

SPECT images of the 3-dimensional brain phantom were also reconstructed using the QSPECT software, following the same procedures as for the clinical scans. The spatial resolution of the reconstructed images was then equalized for these images using recently proposed semi-automated procedures of Hori et al. [32]. Briefly, images obtained for the 3-dimensional brain phantom at each of the three institutions were aligned to the digital design of the phantom structure. Fourier transformation was applied to the both measured and designed images, and the intrinsic spatial resolution or FWHM in units of mm was determined. Referring those FWHM values, Gaussian filter or FWHM that equalizes the spatial resolution was determined by the following equation:

1 $${\text{FWHM}}^{\text{additional}} \, = \,\sqrt {\left( {{\text{FWHM}}^{{{\text{institusion}}\,{\text{B}}}} } \right)^{2} - \,\left( {{\text{FWHM}}^{{{\text{institusion}}\,{\text{A}}}} } \right)^{2} }$$

where FWHM^additional^ represents the FWHM value that equalized the FWHM of SPECT images at the institution B to the FWHM at the institution A which represents the worst spatial resolution, and FWHM^institusion B^ the FWHM at the institution B, and FWHM^institusion A^ the FWHM at the institution A. This additional Gaussian filter was applied to the reconstructed images for clinical data. Functional CBF images were then calculated also for these Gaussian-filtered, spatial resolution-equalized images.

### Data analysis

**Fig. 2:**
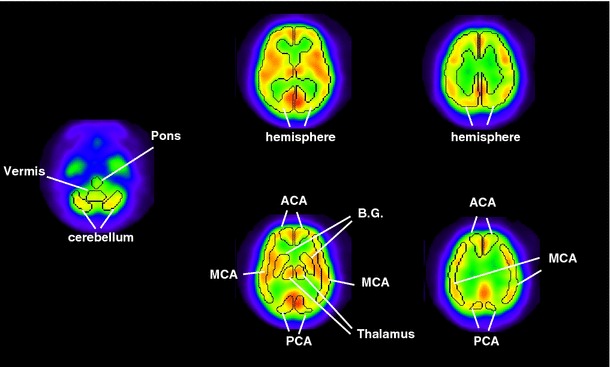
Regions-of-interest (ROI) semi-automatically defined by the NEURO FLEXER software. *ACA* anterior cerebral artery, *MCA* middle cerebral artery, *PCA* posterior cerebral artery, *BG* basal ganglia. Hemisphere includes areas of ACA, MCA, PCA, BG, and thalamus

Regions-of-interest (ROIs) were selected on the spatial resolution-equalized and non-equalized CBF images both at rest and after acetazolamide challenge, using an automatic ROI definition tool, NEURO FLEXER (Nihon Medi-Physics, Tokyo, Japan) [33]. This software automatically transforms the pre-defined three-dimensional ROIs on 16 regions in both hemispheres to individual functional images (Fig. 2). Additional ROIs were placed manually on both hemispheres of the centrum semiovale region (white matter). CBF values were compared between rest and acetazolamide-challenge conditions using paired *t* test. Significant difference in the rest- and acetazolamide-CBF values was evaluated between each of the two combinations of spatial resolution-equalized CBF images using the *t* test and also by Mann–Whitney *U* test. Comparison was also carried out among the three institutions using multiple comparison test (Steel–Dwass method). The analysis of covariance (ANCOVA) was performed for all ROI data, in which the covariates were age, gender, and acetazolamide dosage per weight. Gender difference was also tested for pooled data among the three institutions using Mann–Whitney *U* test.

A three-dimensional stereotaxic surface profile program (3D-SSP, Nihon Medi-Physics, Tokyo, Japan) which is based on the original 3D-SSP program of Minoshima et al. [1, 5] was used to generate four sets of normal database both at rest and after acetazolamide, namely for each of the three institutions and also for the all subjects from all institutions. The statistical difference was then tested using the surface profile of the *Z* score map for each of the four combinations, where *Z* value greater than or equal to 4.53 (*P* < 0.05) was assigned statistically significant [8]. To compare the average value for each pixel according to iSSP3.5-2tZ tool, comparison between two groups was also performed, respectively. This calculation was done for both with and without the spatial resolution equalization.

The CBF images at rest and after acetazolamide administration were globally normalized and transformed into the standard brain atlas using the statistical parametric mapping software (SPM5). The analysis of variance (ANOVA) and the two-sample *t* test were performed with 1000 permutations and a variance smoothing of 10 mm, using the statistical nonparametric mapping (SnPM5), for both spatial resolution-equalized and non-equalized CBF images [34]. The stereotactic coordinates were converted to Talairach space (http://imaging.mrc-cbu.cam.ac.uk/imaging/MniTalairach). *P* < 0.05 (family wise error, FWE) was considered statistically significant. An extent threshold of 100 voxels was applied to judge to be significantly different.

All data are presented as mean ± 1 SD. *P* < 0.05 was considered statistically significant.

## Results

**Fig. 3:**
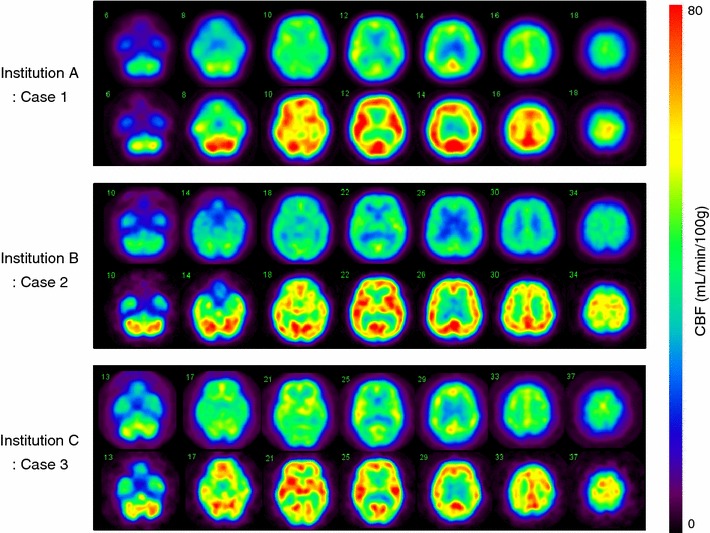
Example clinical images of quantitative CBF before the spatial resolution equalization, at rest and after acetazolamide challenge. One typical case was selected from each of the three institutions. The *same color scale* was applied to these quantitative CBF images

In all institutions, CBF images at rest and after acetazolamide challenge were well obtained. Figure 3 shows typical example images of CBF at rest and after acetazolamide obtained at each of the three institutions. Difference in the spatial resolution among the institutions is apparent.

Table 2 shows the summary results of CBF values obtained from the 3 institutions at rest and after acetazolamide for semi-automatically defined ROIs, by applying the spatial resolution-equalization procedures. Averaged CBF values over entire gray matter regions of all subjects, after the spatial resolution equalization, were 35.7 ± 5.8 and 37.6 ± 6.1 mL/min/100 g at rest, and 51.7 ± 10.3 and 50.9 ± 10.4 mL/min/100 g at after acetazolamide challenge, corresponding to the cortical gray matter region and the cerebellum region, respectively. CVR was 44.9 ± 18.3 % for the whole hemisphere, 46.1 ± 18.2 % in the anterior cerebral artery (ACA) territory, 45.2 ± 18.7 % in the middle cerebral artery (MCA) territory, 48.0 ± 20.8 % in the posterior cerebral artery (PCA) territory, 50.1 ± 19.5 % in the basal ganglia, 40.7 ± 17.5 % in the thalamus, 22.7 ± 16.6 % in the pons, 35.7 ± 18.8 % in the vermis, and 35.2 ± 17.2 % in the cerebellum regions. CBF values were significantly greater after acetazolamide challenge than those at rest in all ROIs in all institutions. The *t* test and Mann–Whitney’s *U* test did not detect any significant differences at rest in each of institution combinations, but after acetazolamide challenges they demonstrated significant differences in the bilateral PCA and MCA territories, for the institutions A and B, and also significant differences in the bilateral PCA territories for the institutions A and C. The multiple comparison analysis, on the other hand, demonstrated no significant difference in CBF both at rest and after acetazolamide challenge, except for the left PCA area only after acetazolamide challenge between A and B institutions. The ANCOVA also resulted in no significant dependency of CBF values in terms of the age or the effective dose of acetazolamide. The Mann–Whitney *U* test demonstrated significant gender-dependent difference (greater CBF values in female than in male) for the data pooled among the three institutions at the hemisphere: ACA, MCA, basal ganglia, thalamus, and cerebellum regions at rest. The significant gender-dependent difference was also seen in the institution B at rest in the areas of hemisphere: ACA, MCA, PCA, basal ganglia, thalamus and the white matter. No significant difference was observed after acetazolamide challenge in any institutions or in the pooled data.

**Table 2:** Summary results of CBF values obtained after spatial resolution-equalized CBF images at rest and after acetazolamide challenge

Rest	Institution A (*n* = 9)	Institution B (*n* = 13)	Institution C (*n* = 10)	All institutions (*n* = 32)
	Mean ± SD (mL/min/100 g)	Mean ± SD (mL/min/100 g)	Mean ± SD (mL/min/100 g)	Mean ± SD (mL/min/100 g)
Hemisphere				
Right	37.6 ± 6.1	34.2 ± 6.4	36.4 ± 4.2	35.9 ± 5.9
Left	37.2 ± 6.2	34.1 ± 6.4	36.2 ± 3.9	35.6 ± 5.8
Anterior cerebral artery (ACA)				
Right	38.8 ± 6.5	34.4 ± 7.6	36.2 ± 4.7	36.2 ± 6.7
Left	38.0 ± 6.2	34.4 ± 7.3	36.7 ± 4.7	36.1 ± 6.5
Middle cerebral artery (MCA)				
Right	38.5 ± 6.3	34.4 ± 6.6	36.6 ± 4.2	36.3 ± 6.1
Left	37.5 ± 6.1	33.9 ± 6.5	36.0 ± 3.9	35.6 ± 5.9
Posterior cerebral artery (PCA)				
Right	42.7 ± 7.2	37.5 ± 6.7	38.7 ± 4.4	39.3 ± 6.6
Left	42.8 ± 7.9	37.4 ± 6.4	38.6 ± 3.8	39.3 ± 6.6
Basal ganglia (BG)				
Right	39.4 ± 6.1	36.9 ± 6.7	39.4 ± 4.9	38.4 ± 6.1
Left	39.0 ± 5.7	37.0 ± 7.1	39.1 ± 4.3	38.2 ± 6.1
Thalamus				
Right	36.0 ± 5.1	34.0 ± 6.2	37.5 ± 3.4	35.7 ± 5.4
Left	35.7 ± 5.0	34.4 ± 6.0	37.8 ± 3.7	35.8 ± 5.3
Cerebellum				
Right	38.6 ± 6.3	36.6 ± 6.4	37.3 ± 4.1	37.4 ± 5.8
Left	39.8 ± 6.8	36.4 ± 7.0	38.2 ± 4.6	37.9 ± 6.4
Centrum semiovale (white matter)				
Right	26.8 ± 4.0	23.9 ± 4.5	27.5 ± 3.5	25.8 ± 4.4
Left	26.5 ± 4.7	24.0 ± 4.2	27.1 ± 3.4	25.7 ± 4.3
Averaged for right and left				
Hemisphere	37.4 ± 6.1	34.2 ± 6.4	36.3 ± 4.0	35.7 ± 5.8
ACA	38.4 ± 6.3	34.4 ± 7.5	36.5 ± 4.7	36.2 ± 6.6
MCA	38.0 ± 6.2	34.2 ± 6.5	36.3 ± 4.0	35.9 ± 6.0
PCA	42.7 ± 7.4	37.4 ± 6.5	38.7 ± 4.0	39.3 ± 6.5
BG	39.2 ± 5.8	36.9 ± 6.9	39.2 ± 4.6	38.3 ± 6.1
Thalamus	35.9 ± 4.9	34.2 ± 6.1	37.7 ± 3.5	35.8 ± 5.3
Pons	30.7 ± 6.2	29.3 ± 5.2	31.5 ± 3.5	30.4 ± 5.1
Vermis	41.1 ± 7.3	36.8 ± 6.4	40.4 ± 4.1	39.1 ± 6.4
Cerebellum	39.2 ± 6.5	36.5 ± 6.6	37.7 ± 4.3	37.6 ± 6.1
White matter	26.7 ± 4.3	24.0 ± 4.3	27.3 ± 3.4	25.8 ± 4.3

Acetazolamide	Institution A	Institution B	Institution C	All institutions
	Mean ± SD (mL/min/100 g)	Mean ± SD (mL/min/100 g)	Mean ± SD (mL/min/100 g)	Mean ± SD (mL/min/100 g)
Hemisphere				
Right	58.6 ± 10.6	49.3 ± 9.3	49.7 ± 9.2	52.1 ± 10.5
Left	57.5 ± 10.7	48.7 ± 8.7	49.2 ± 8.8	51.3 ± 10.1
Anterior cerebral artery (ACA)				
Right	60.0 ± 11.2	50.1 ± 11.0	49.9 ± 9.7	52.8 ± 11.5
Left	59.3 ± 11.5	49.9 ± 10.0	50.6 ± 9.4	52.8 ± 11.1
Middle cerebral artery (MCA)				
Right	60.4 ± 10.6	49.4 ± 9.4	50.1 ± 9.7	52.7 ± 11.0
Left	58.6 ± 10.6	48.2 ± 8.9	49.3 ± 8.8	51.5 ± 10.4
Posterior cerebral artery (PCA)				
Right	68.9 ± 15.4	54.6 ± 10.3	54.4 ± 9.5	58.6 ± 13.4
Left	69.2 ± 15.8^††^	53.5 ± 9.0^††^	53.6 ± 9.6	57.9 ± 13.5
Basal ganglia (BG)				
Right	64.0 ± 11.1	55.7 ± 10.6	54.8 ± 11.1	57.7 ± 11.6
Left	62.2 ± 9.8	56.0 ± 11.0	53.6 ± 10.0	57.0 ± 10.9
Thalamus				
Right	53.9 ± 8.0	48.5 ± 8.4	49.7 ± 9.3	50.4 ± 8.9
Left	53.4 ± 9.6	48.1 ± 7.6	49.1 ± 9.3	49.9 ± 9.0
Cerebellum				
Right	54.2 ± 12.5	50.2 ± 9.6	46.6 ± 6.9	50.2 ± 10.2
Left	57.1 ± 11.8	49.9 ± 10.6	48.8 ± 8.4	51.6 ± 10.9
Centrum semiovale (white matter)				
Right	36.8 ± 6.0	32.4 ± 5.8	35.5 ± 7.2	34.6 ± 6.6
Left	37.0 ± 6.5	31.3 ± 5.2	34.6 ± 7.2	33.9 ± 6.7
Averaged for right and left				
Hemisphere	58.1 ± 10.7	49.0 ± 9.0	49.5 ± 9.0	51.7 ± 10.3
ACA	59.6 ± 11.3	50.0 ± 10.5	50.3 ± 9.5	52.8 ± 11.3
MCA	59.5 ± 10.6	48.8 ± 9.1	49.7 ± 9.2	52.1 ± 10.7
PCA	69.0 ± 15.5	54.1 ± 9.6	54.0 ± 9.4	58.2 ± 13.3
BG	63.1 ± 10.4	55.8 ± 10.8	54.2 ± 10.5	57.4 ± 11.2
Thalamus	53.7 ± 8.8	48.3 ± 7.9	49.4 ± 9.2	50.2 ± 8.9
Pons	37.3 ± 9.6	37.3 ± 6.4	36.8 ± 6.5	37.2 ± 7.4
Vermis	60.1 ± 14.0	50.3 ± 9.4	50.3 ± 8.3	53.0 ± 11.5
Cerebellum	55.6 ± 11.8	50.0 ± 10.0	47.7 ± 7.5	50.9 ± 10.4
White matter	36.9 ± 6.1	31.9 ± 5.4	35.1 ± 7.2	34.3 ± 6.6

Values are mean ± 1 standard deviation (SD)

^††^ *P* = 0.04

**Fig. 4:**
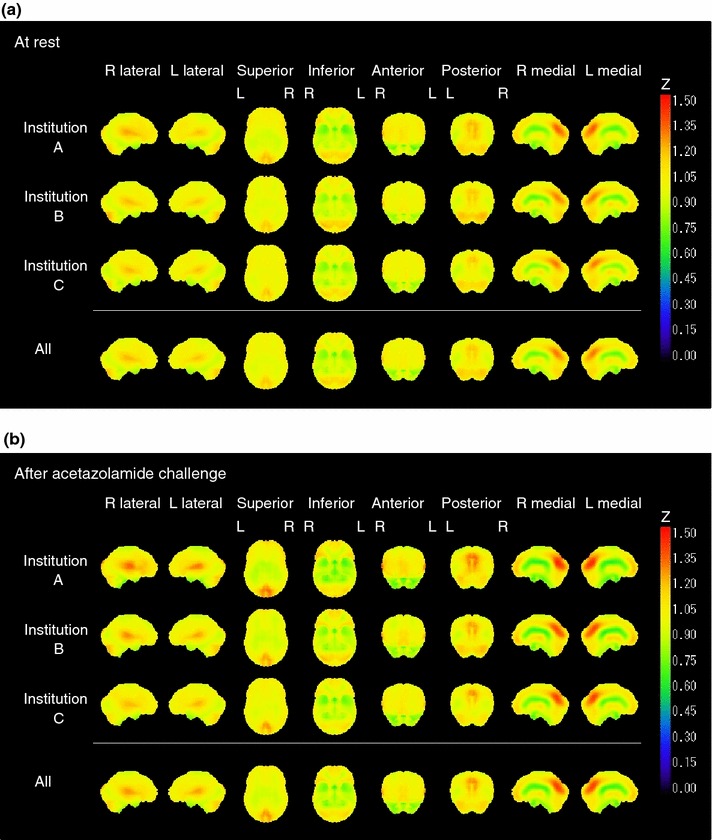
Stereotactic CBF images, normalized and averaged over the subjects at each institution and also for all subjects, at rest (**a**) and after acetazolamide challenge (**b**). Calculation was performed on spatial resolution-equalized images using 3D-SSP software. No region showed statistical difference between any combinations of the institutions, after the spatial resolution-equalization procedure

Figure 4 shows results from the 3D-SSP software of CBF images both at rest and after acetazolamide challenge, in which the spatial resolution-equalized CBF images were utilized. No regions were shown to represent significant difference among the three institutions. When non-equalized CBF images were equalized, there was significant difference in a few pixels, in the thalamus and the posterior part of the cortex regions.

**Fig. 5:**
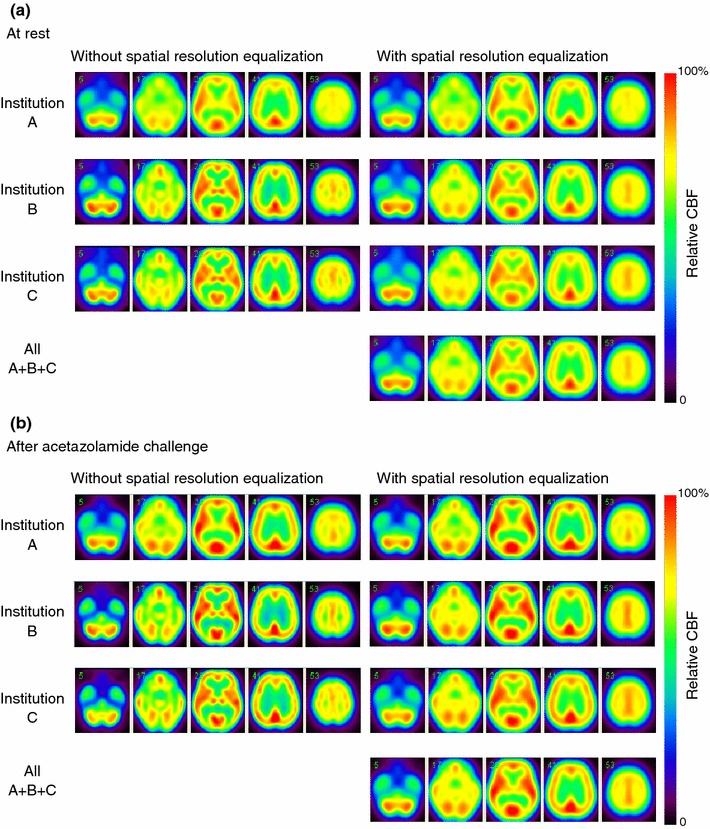
Comparison of CBF images normalized over the subjects at each institution at rest (**a**) and after acetazolamide challenge (**b**). Spatial resolution-equalized CBF images were used for this analysis. Statistical analysis demonstrated that no significance was seen among the three institutions both at rest and after acetazolamide challenge, when the extent threshold of 100 voxels was selected. Note that all images are displayed using the *same color scale* with the normalized maximum values at each condition

Figure 5 shows results from the SPM5 analysis, demonstrating rest- and acetazolamide-CBF images averaged over the subjects at each of three institutions, with and without the spatial resolution-equalization procedures. Images before applying the spatial resolution equalization demonstrated poorer spatial resolution in the institution A than others. Small differences are visible among the three institutions particularly in the thalamus and semiovale center regions, but the spatial equalization resulted in reduced differences among institutions. The two-sample *t* test with applying the spatial resolution-equalized CBF images detected significant differences at rest, in regions of the medial frontal gyrus at rest for institution A vs. B (*x* = 4, *y* = 29, *z* = −10; *k* = 152 voxels), and the right fusiform gyrus (*x* = 38, *y* = −39, *z* = −11; *k* = 656 voxels) and the midbrain (*x* = 2, *y* = −27, *z* = −5; *k* = 534 voxels) after acetazolamide for institution A vs. C, and a region of the right fusiform gyrus (*x* = 34, *y* = −41, *z* = −6; *k* = 293 voxels) for institution B vs. C. Without the application of the spatial resolution equalization, significant difference was seen in various regions including the left claustrum at rest for the institution A vs. B or A vs. C, and in the temporal gyrus and the thalamus after acetazolamide for the institution A vs. B or A vs. C. The ANOVA analysis also detected differences in regions of the left claustrum (*x* = −34, *y* = −10, *z* = 2; *k* = 109 voxels) and in the right inferior frontal gyrus at rest (*x* = 48, *y* = 16, *z* = 12; *k* = 50 voxels), and in a region of the right medial frontal gyrus after acetazolamide (*x* = 46, *y* = 6, *z* = 44; *k* = 16 voxels) at rest, when the spatial resolution equalization is not applied. When the spatial resolution equalization was applied, differences were seen in a region of in the left insula (*x* = −40, *y* = −12, *z* = 2; *k* = 11 voxels) at rest and in a region of the right medial temporal gyrus (*x* = 51, *y* = −44, *z* = 4; *k* = 18 voxels) after acetazolamide challenge. Of note is that applying the extent threshold of 100 voxels according to the criteria defined in this study vanished the differences, and no regions indicated significant difference.

## Discussion

In this study, we demonstrated the inter-institutional agreement of quantitative CBF value at rest and after acetazolamide challenge on healthy subjects obtained independently at three different institutions equipped with different SPECT devices. Particular attention made was to standardize the detailed operational procedures during the SPECT scanning by following the previously validated dual-administration autoradiography protocol by means of dual-administration of ^123^I-IMP, and the standardized SPECT reconstruction using the QSPECT package. It was shown that quantitative CBF values and the relative distribution of CBF images appeared to be in a reasonable agreement among the three institutions, on the stereotactic domain both at rest and after acetazolamide challenge. Differences, which were observed when CBF images were simply pooled without adjusting the spatial resolution, vanished by equalizing the spatial resolution at each SPECT scanner. Findings of (a) no significant difference in regional CBF values in all ROIs both at rest and after acetazolamide challenge except for the PCA region after acetazolamide, as well as (b) no significant differences in all pixels both at rest and after acetazolamide after spatial resolution equalization suggest that application of computer-aided diagnosis-assisting tools such as the 3D-SSP and SPM software programs may be valid to SPECT images, when the quantitative reconstruction program shown in this study (QSPECT) is employed. A database acquired at one of the institutions presented in this study may therefore be shared among clinical institutions which likely do not have their own database. This would be an advantage of using SPECT in routine clinical studies, because this has not been achieved in PET due to the fact that the scatter and other physical factors related to the counting rate performances are significantly dependent on the scanner geometric design and electric circuit.

Ischemic status in patients with occlusion or stenosis in their middle cerebral arteries can be well characterized by quantitative regional CBF [11, 35] and CVR in response to acetazolamide challenge [36–38]. It was also demonstrated that the evaluation of those functional parameters provides prognosis after revascularization therapies [39]. Semi-automatic discrimination of ischemic severity from Stage-0 to Stage-II using computer-aided diagnosis-supporting tools such as SEEJET software [5] may therefore be relevant, if the SPECT images are reconstructed using a single program for quantitative SPECT reconstruction such as QSPECT.

Regional CBF values in the cortical gray matter regions (35.7 ± 5.8 mL/min/100 g) obtained in this study were consistent with the values obtained in our earlier work [12] using essentially the same technique on young healthy adults (36.2 ± 8.2 mL/min/100 g), and also those measured using PET on healthy volunteers (39.4 ± 7.3 mL/min/100 g) [40]. The CBF values at rest were however larger than those obtained by the same procedures with ^123^I-IMP but using a SPECT reconstruction supplied from a vendor of the SPECT device by Hatazawa et al. [35] in cortical gray matter regions in normals (33.0 ± 5.1 mL/min/100 g). This work employed the correction for attenuation (with a broad *μ* value) but not for the scatter. As demonstrated in earlier studies, the image contrast was reduced by a factor of 20 % [12], if the scatter correction is not applied [41, 42], and the attenuation correction was made using a broad *μ* value. The inter-subject variation of CBF values of approximately 16 % was consistent as compared to the previously reported values assessed in a multicenter study using ^15^O-water and PET [43] and other reports on healthy subjects [12, 35, 40, 44]. These also support the quality of the normal database presented in this study.

The CBF values obtained in this study were on the other hand smaller than those measured using ^15^O-water and PET. A multicenter study by Ito et al. [43] involving 11 institutions resulted in CBF at rest being 44.4 ± 6.5 mL/min/100 g in the cortical gray matter regions. Yamaguchi et al. [44] reported CBF at rest 43.0 ± 7.0 mL/min/100 g in an aged healthy volunteer group. A recent work of direct comparison in a patient population also demonstrated the SPECT-CBF with the DTARG protocol being systematically smaller than that by the ^15^O-water PET by a factor of approximately 10 % [13]. This difference could partly be explained by the fact that the first-pass extraction fraction of ^123^I-IMP is smaller as compared to that of ^15^O-water [11, 13]. Another factor could be attributed to the poorer spatial resolution of SPECT as compared to PET [45]. It should also be noted that the quantitative CBF values depend on the intrinsic spatial resolution of reconstructed SPECT images, and thus can vary dependent on the collimator specification. Since this collimator-dependent spatial resolution has not been taken into account in the reconstruction software, the spatial resolution equalization has been implemented in the grouped analysis in this study, by means of the post Gaussian filtering operation before the stereotactic statistical analysis. It appeared to be essential to provide quantitative CBF images which are consistent among the scanners installed at different institutions.

Increase in CBF after acetazolamide challenge, or CVR, was approximately 40 % in this study, which is also consistent with earlier reports [13, 37, 46]. Of these, Hayashida et al. [46] showed that CBF increase was 41.4 ± 16.3 % at 10 min after the acetazolamide challenge, and then reduced to 27.7 ± 15.3 % after 20 min. Earlier works [14, 47] demonstrated that the observed CBF with this technique (IMPARG and DTARG) is not a simple average over the total scan duration, but is weighted by transiently changing contribution factors. This contribution factor was approximately proportional to the first derivative of the tissue time-activity curve in the intravenous ^123^I-IMP study, and thus is highly weighted during the first 2–3 min after IMP administration. This suggests that the acetazolamide CBF presented in this study likely corresponds to the peak values. The statistical analysis for the data pooled among the three institutions demonstrated that CBF values were greater in female than in male at rest in the hemisphere: ACA, MCA, basal ganglia, thalamus, and cerebellum regions. The significant gender-dependent difference was also seen in the institution B at rest in the areas of hemisphere: ACA, MCA, PCA, basal ganglia, thalamus and the white matter. The greater CBF in female than male is consistent with previous findings [48]. It should however be noted that no significant difference was observed after acetazolamide challenge in any institutions, or in the pooled data.

No obvious difference was seen in relative distributions of CBF in gray matter regions between rest and acetazolamide. Consequently, there was no significant difference in the CVR in all regions. Ito et al. [49] demonstrated that using the ^15^O-water PET technique there were several gray matter areas which represented smaller amount of increases in CBF during the carbon-dioxide inhalation, such as in the pons, cingulate gyrus, temporal cortex, tempo-occipital cortex, and occipital cortex regions. They then claimed that those areas had different (smaller) vascular reactivity to CO_2_ compared with other areas. The discrepancy with the present study could be due to different vaso-dilating mechanisms of acetazolamide compared with carbon dioxide. One also might consider methodological factors such as (a) the poorer spatial resolution of SPECT compared with PET, (b) systematic errors in the CBF calculation processes, or (c) the imperfect assumption of model parameters such as the brain-to-blood partition coefficient or distribution volume for ^15^O-water or ^123^I-IMP, and (d) the contribution of arterial blood volume. Distribution volume is greater for ^123^I-IMP than for ^15^O-water, which causes slower kinetics for ^123^I-IMP than those for ^15^O-water, resulting in enhanced sensitivity to various error factors [21, 45]. Errors associated with the blood volume should also be smaller for ^123^I-IMP than that for ^15^O-water, because of greater signals from tissue relative to the blood radioactivity in ^123^I-IMP than in ^15^O-water.

There are a couple issues which need to be addressed. There was a significant difference in CBF in the PCA area (Table 2) between institutions A and B after acetazolamide challenge. This might be partly attributed to the global difference caused by the larger administration dose if normalized by the body weight (Table 1). Another reason could be due to different design of the head rest, which requires further investigation. It should also be addressed that administration dosage of acetazolamide per body weight, ^123^I-IMP dose, and time of the day at which the study was conducted was significantly different among the three institutions. Effects of these factors are however likely negligible. The healthy subjects with an age of 61.7 ± 3.8 were selected in this study. The DTARG technique has often been applied to patients with Moyamoya disease who are often younger than the population in this study. A study needs to be carried out to generate a normal data base in younger populations. 3D-SSP and eZIS software programs have been utilized to spatially normalize the local distribution in this study. However, the adequacy and accuracy of these programs are yet to be verified in diseased areas. One major source of errors was subjects’ movements during the scan. Because of a relatively long study duration (approximately 1 h for the whole procedure), it is likely that some subjects moved, particularly after the acetazolamide challenge. A software program has been implemented to align the last 30-min images to the initial images, as described in earlier study [13]. Further technique may need to be developed to compensate for the movement during the scan.

The equipment-dependent differences of reconstructed images are essential limitations when one intends to utilize SPECT or PET devices in clinical multicenter trials. Several phantoms have been employed previously to compensate the scanner-dependent differences. Use of 3D-Hoffman brain phantom is an example, and it has been used in a multicenter SPECT study to compensate the scanner-specific distortion of the reconstructed images [8]. Striatum phantom was employed to calibrate the striatum-to-background counts ratios in SPECT images among different institutions for ^123^I-FP-CIT [50]. Joshi et al. [51] also employed 3D-Hoffman phantom filled with ^18^F-solution to reduce the scanner-specific distortion of images in a multicenter study that employed PET scanners. It has however been shown that adjustment works only for the high-frequency component, but not for the low-frequency component which is associated with the intrinsic errors in the scatter and attenuation correction procedures [51]. The reason for this was explained by the different shape of 3D-Hoffman phantom from the human brain, resulting in different magnitude and distribution of errors, suggesting the essential limitation of using the 3D-Hoffman brain phantom for this purpose. The scanner-dependent variation in regard to the attenuation and scatter essentially does not exist in SPECT, if appropriate SPECT reconstruction technique is employed.

## Conclusion

Application of the recently developed QSPECT software package to a dual-administration ^123^I-IMP SPECT scan with a carefully designed protocol provided quantitative CBF images at rest and after acetazolamide challenge on healthy volunteers, which were consistent among the participating three institutions, in terms of the quantitative values and the regional distribution of CBF. Use of a common database may therefore be feasible when one intends to use a computer-assisted diagnostic software tool for defining the ischemic stages in patients with stenosis and/or occlusion in their major arteries.

## References

[1] Minoshima S, Frey KA, Koeppe RA, Foster NL, Kuhl DE (1995). A diagnostic approach in Alzheimer’s disease using three-dimensional stereotactic surface projections of fluorine-18-FDG PET. J Nucl Med.

[2] Ishii K, Kono AK, Sasaki H, Miyamoto N, Fukuda T, Sakamoto S (2006). Fully automatic diagnostic system for early- and late-onset mild Alzheimer’s disease using FDG PET and 3D-SSP. Eur J Nucl Med Mol Imaging.

[3] Minoshima S, Giordani B, Berent S, Frey KA, Foster NL, Kuhl DE (1997). Metabolic reduction in the posterior cingulate cortex in very early Alzheimer’s disease. Ann Neurol.

[4] Mizumura S, Kumita S, Cho K, Ishihara M, Nakajo H, Toba M (2003). Development of quantitative analysis method for stereotactic brain image: assessment of reduced accumulation in extent and severity using anatomical segmentation. Ann Nucl Med.

[5] Mizumura S, Nakagawara J, Takahashi M, Kumita S, Cho K, Nakajo H (2004). Three-dimensional display in staging hemodynamic brain ischemia for JET study: objective evaluation using SEE analysis and 3D-SSP display. Ann Nucl Med.

[6] Nakagawara J, Hyogo T, Kataoka T, Hayase K, Kasuya J, Kamiyama K (2000). Role of neuroimaging (SPECT/PET, CT/MRI) in thrombolytic therapy. No To Shinkei.

[7] Nakagawara J (1999). Clinical neuroimaging of cerebral ischemia. No To Shinkei.

[8] Matsuda H, Mizumura S, Nagao T, Ota T, Iizuka T, Nemoto K (2007). Automated discrimination between very early Alzheimer disease and controls using an easy *Z*-score imaging system for multicenter brain perfusion single-photon emission tomography. AJNR Am J Neuroradiol.

[9] Friston KJ. Analyzing brain images: principles and overview. In: Frackowiak RSJ, Friston KJ, Frith C, Dolan R, Mazziotta JC, editors. Human brain function. USA: Academic; 1997. p. 25-41.

[10] Hapdey S, Soret M, Ferrer L, Koulibaly P, Henriques J, Bardiès M (2004). Quantification in SPECT: myth or reality? A multicentric study. IEEE Nucl Sci Symp Conf Record.

[11] Iida H, Akutsu T, Endo K, Fukuda H, Inoue T, Ito H (1996). A multicenter validation of regional cerebral blood flow quantitation using [^123^I]iodoamphetamine and single photon emission computed tomography. J Cereb Blood Flow Metab.

[12] Iida H, Narita Y, Kado H, Kashikura A, Sugawara S, Shoji Y (1998). Effects of scatter and attenuation correction on quantitative assessment of regional cerebral blood flow with SPECT. J Nucl Med.

[13] Iida H, Nakagawara J, Hayashida K, Fukushima K, Watabe H, Koshino K (2010). Multicenter evaluation of a standardized protocol for rest and acetazolamide cerebral blood flow assessment using a quantitative SPECT reconstruction program and split-dose ^123^I-iodoamphetamine. J Nucl Med.

[14] Kim KM, Watabe H, Hayashi T, Hayashida K, Katabuchi T, Enomoto N (2006). Quantitative mapping of basal and vasareactive cerebral blood flow using split-dose ^123^I-iodoamphetamine and single photon emission computed tomography. Neuroimage.

[15] Yoneda H, Shirao S, Koizumi H, Oka F, Ishihara H, Ichiro K (2012). Reproducibility of cerebral blood flow assessment using a quantitative SPECT reconstruction program and split-dose ^(123)^I-iodoamphetamine in institutions with different gamma-cameras and collimators. J Cereb Blood Flow Metab.

[16] Graham LS, Fahey FH, Madsen MT, van Aswegen A, Yester MV (1995). Quantitation of SPECT performance: report of Task Group 4, Nuclear Medicine Committee. Med Phys.

[17] Kim KM, Watabe H, Shidahara M, Ishida Y, Iida H (2001). SPECT collimator dependency of scatter and validation of transmission dependent scatter compensation methodologies. IEEE Trans Nucl Sci.

[18] Iida H, Itoh H, Nakazawa M, Hatazawa J, Nishimura H, Onishi Y (1994). Quantitative mapping of regional cerebral blood flow using iodine-123-IMP and SPECT. J Nucl Med.

[19] Iida H, Hori Y, Ishida K, Imabayashi E, Matsuda H, Takahashi M, et al. Three-dimensional brain phantom containing bone and grey matter structures with a realistic head contour. Ann Nucl Med. 2013;27(1):25–36. 10.1007/s12149-012-0655-7 PMC3549246 23011903

[20] Iida H, Itoh H, Bloomfield PM, Munaka M, Higano S, Murakami M (1994). A method to quantitate cerebral blood flow using a rotating gamma camera and iodine-123 iodoamphetamine with one blood sampling. Eur J Nucl Med Mol Imaging.

[21] Iida H, Nakazawa M, Uemura K (1995). Quantitation of regional cerebral blood flow using ^123^I-IMP from a single SPECT scan and a single blood sampling—analysis on statistical error source and optimal scan time. Kaku Igaku.

[22] Kurisu R, Ogura T, Takikawa S, Saito H, Nakazawa M, Iida H (2002). Estimation and optimization of the use of standard arterial input function for split-dose administration of *N*-isopropyl-*p*[^123^I]iodoamphetamine. Kaku Igaku.

[23] Ogura T, Takikawa S, Saito H, Nakazawa M, Shidahara M, Iida H (1999). Validation and optimization of the use of standardized arterial input function in *N*-isopropyl-*p*[^123^I]iodoamphetamine cerebral blood flow SPECT. Kaku Igaku.

[24] Iida H, Eberl S (1998). Quantitative assessment of regional myocardial blood flow with thallium-201 and SPECT. J Nucl Cardiol.

[25] Kim KM, Varrone A, Watabe H, Shidahara M, Fujita M, Innis RB (2003). Contribution of scatter and attenuation compensation to SPECT images of nonuniformly distributed brain activities. J Nucl Med.

[26] Narita Y, Eberl S, Iida H, Hutton BF, Braun M, Nakamura T (1996). Monte Carlo and experimental evaluation of accuracy and noise properties of two scatter correction methods for SPECT. Phys Med Biol.

[27] Narita Y, Iida H, Eberl S, Nakamura T (1997). Monte Carlo evaluation of accuracy and noise properties of two scatter correction methods for ^201^Tl cardiac SPECT. IEEE Trans Nucl Sci.

[28] Eberl S, Kanno I, Fulton RR, Ryan A, Hutton BF, Fulham MJ (1996). Automated interstudy image registration technique for SPECT and PET. J Nucl Med.

[29] Meikle SR, Hutton BF, Bailey DL (1994). A transmission-dependent method for scatter correction in SPECT. J Nucl Med.

[30] Iida H, Shoji Y, Sugawara S, Kinoshita T, Tamura Y, Narita Y (1999). Design and experimental validation of a quantitative myocardial ^201^Tl SPECT system. IEEE Trans Nucl Sci.

[31] Narita Y, Iida H (1999). Scatter correction in myocardial thallium SPECT: needs for optimization of energy window settings in the energy window-based scatter correction techniques. Kaku Igaku.

[32] Hori Y, Zeniya T, Yamamoto A, Koshino K, Enmi J, Iguchi S, et al., editors. Novel method for simultaneous evaluation of spatial resolution and image accuracy using three dimensional brain phantom—application to a SPECT multicenter study. J Nucl Med. 2012;141.

[33] Ogura T, Hida K, Masuzuka T, Saito H, Minoshima S, Nishikawa K (2009). An automated ROI setting method using NEUROSTAT on cerebral blood flow SPECT images. Ann Nucl Med.

[34] Nichols TE, Holmes AP (2002). Nonparametric permutation tests for functional neuroimaging: a primer with examples. Hum Brain Mapp.

[35] Hatazawa J, Iida H, Shimosegawa E, Sato T, Murakami M, Miura Y (1997). Regional cerebral blood flow measurement with iodine-123-IMP autoradiography: normal values, reproducibility and sensitivity to hypoperfusion. J Nucl Med.

[36] Ogasawara K, Ogawa A, Yoshimoto T (2002). Cerebrovascular reactivity to acetazolamide and outcome in patients with symptomatic internal carotid or middle cerebral artery occlusion: a xenon-133 single-photon emission computed tomography study. Stroke.

[37] Ogasawara K, Ogawa A, Terasaki K, Shimizu H, Tominaga T, Yoshimoto T (2002). Use of cerebrovascular reactivity in patients with symptomatic major cerebral artery occlusion to predict 5-year outcome: comparison of xenon-133 and iodine-123-IMP single-photon emission computed tomography. J Cereb Blood Flow Metab.

[38] Ogasawara K, Ito H, Sasoh M, Okuguchi T, Kobayashi M, Yukawa H (2003). Quantitative measurement of regional cerebrovascular reactivity to acetazolamide using ^123^I-*N*-isopropyl-*p*-iodoamphetamine autoradiography with SPECT: validation study using H_2_^15^O with PET. J Nucl Med.

[39] Ogasawara K, Ogawa A (2006). JET study (Japanese EC-IC Bypass Trial). Nippon Rinsho.

[40] Iida H, Miura S, Shoji Y, Ogawa T, Kado H, Narita Y (1998). Noninvasive quantitation of cerebral blood flow using oxygen-15–water and a dual-PET system. J Nucl Med.

[41] Iida H, Takahashi M, Motomura N, Hachiya T, Nakagawara J (1996). Effects of Compton scatter in quantitative brain SPECT. Kaku Igaku.

[42] Zaidi H, Koral KF (2004). Scatter modelling and compensation in emission tomography. Eur J Nucl Med Mol Imaging.

[43] Ito H, Kanno I, Kato C, Sasaki T, Ishii K, Ouchi Y (2004). Database of normal human cerebral blood flow, cerebral blood volume, cerebral oxygen extraction fraction and cerebral metabolic rate of oxygen measured by positron emission tomography with ^15^O-labelled carbon dioxide or water, carbon monoxide and oxygen: a multicentre study in Japan. Eur J Nucl Med Mol Imaging.

[44] Yamaguchi T, Kanno I, Uemura K, Shishido F, Inugami A, Ogawa T (1986). Reduction in regional cerebral metabolic rate of oxygen during human aging. Stroke.

[45] Iida H, Narita Y, Ardekani BA, Hatazawa J, Nakazawa M, Kanno I (1995). Evaluation of partial volume effect in quantitative measurement of regional cerebral blood flow in single photon emission computed tomography—effects of limited spatial resolution and first-pass extraction fraction. Kaku Igaku.

[46] Hayashida K, Tanaka Y, Hirose Y, Kume N, Iwama T, Miyake Y (1996). Vasoreactive effect of acetazolamide as a function of time with sequential PET ^15^O-water measurement. Nucl Med Commun.

[47] Iida H, Kanno I, Miura S. Rapid measurement of cerebral blood flow with positron emission tomography. In: Chadwick DJ, Whelan J, editors. Exploring the brain functional anatomy with positron tomography. Chichester: Wiley; 1991. p. 23–42. 1815893

[48] Hatazawa J, Iida H, Shimosegawa E, Sato T, Murakami M, Miura Y (1997). Regional cerebral blood flow measurement with iodine-123-IMP autoradiography: normal values, reproducibility and sensitivity to hypoperfusion. J Nucl Med.

[49] Ito H, Yokoyama I, Iida H, Kinoshita T, Hatazawa J, Shimosegawa E (2000). Regional differences in cerebral vascular response to PaCO_2_ changes in humans measured by positron emission tomography. J Cereb Blood Flow Metab.

[50] Tossici-Bolt L, Dickson JC, Sera T, de Nijs R, Bagnara MC, Jonsson C (2011). Calibration of gamma camera systems for a multicentre European ^(1)(2)(3)^I-FP-CIT SPECT normal database. Eur J Nucl Med Mol Imaging.

[51] Joshi A, Koeppe RA, Fessler JA (2009). Reducing between scanner differences in multi-center PET studies. Neuroimage.

